# Sex Differences in Ependymoma Methylation by Methylation‐Defined Subgroup

**DOI:** 10.1111/jcmm.70286

**Published:** 2024-12-16

**Authors:** Shelby Mestnik, Natali Sorajja, Zhanni Lu, Lauren J. Mills, Lindsay Williams

**Affiliations:** ^1^ Pediatric Hematology and Oncology, Department of Pediatrics University of Minnesota Minneapolis Minnesota USA; ^2^ Division of Epidemiology & Clinical Research, Department of Pediatrics University of Minnesota Minneapolis Minnesota USA; ^3^ Albert Einstein College of Medicine Bronx New York USA; ^4^ Masonic Cancer Center University of Minnesota Minneapolis Minnesota USA; ^5^ Brain Tumor Program University of Minnesota Minneapolis Minnesota USA

## Abstract

Ependymoma is the second most common malignant paediatric brain tumour composed of nine methylation‐defined, clinically relevant subgroups. It is unclear if there are sex differences in methylation profiles within these subgroups which could guide future treatment options. We obtained available methylation data from the National Center for Biotechnology Information Gene Expression Omnibus (GEO). Differentially methylated probes (DMPs) between sexes were identified in each ependymoma sample and mapped to genes. Reactome pathways resulting from genes were identified. Survival was estimated for each sex within molecular subgroups. There were 492 cases included in the main analysis: PF‐EPN‐A (*n* = 238) PF‐EPN‐B (*n* = 52), PF‐SE (*n* = 34), SP‐MPE (*n* = 26), SP‐EPN (*n* = 21), ST‐EPN‐RELA (*n* = 87), ST‐EPN‐YAP1 (*n* = 13) and ST‐SE (*n* = 21). Females were observed to have better, but statistically nonsignificant, 5‐year overall survival (OS) and better, marginally significant 5‐year progression‐free survival (PFS) than males. One subgroup, ST‐EPN‐RELA, showed significantly better OS in females. There was a difference in immune cell composition within tumour subgroups. One gene, RFTN1, was consistently differentially methylated by sex among all subgroups. There were biologic pathways identified from genes with differential methylation by sex in the following subgroups: PF‐EPN‐B, PF‐SE, ST‐EPN‐RELA and ST‐EPN‐YAP1. Many of the identified pathways may be options for potential therapeutic targets.

## 1 Introduction

Ependymoma is the third most common paediatric brain tumour and the second most common malignant paediatric brain tumour [1, 2, 3, 4]. On average, it affects 200 children in the United States annually [5] and accounts for around 10% of brain tumours in children [3]. Historically, ependymal tumours were classified by histopathology regardless of location [4], which was of little use in predicting tumour behaviour [3, 6]. More recently, clinically relevant and prognostic classification methods have been prioritised and, now using methylation data, nine subgroups have been identified [3, 6, 7]. These subgroups are found among three different anatomical areas, including supratentorial (ST), posterior fossa (PF) and the spinal cord [3]. Subgroups include three different entities within each anatomic compartment [8]. For example, supratentorial ependymomas are associated with RELA (poor outcome) or YAP fusions (better outcomes) [4], whereas adults generally have spinal tumours associated with changes in the NF2 gene and tumours are of the SP‐MPE subgroup [7]. Overall survival (OS) is around 60% in paediatric patients [7, 8], but progression‐free survival (PFS) is much lower, around 20%–50% depending on the methylation‐defined subgroup [2, 6]. How these outcomes vary by sex has been less frequently explored in the paediatric population.

There are distinct sex differences in ependymoma incidence such that males have an estimated 23% excess in incidence compared to females in the paediatric population [9]. Additionally, we have reported on sex differences in ependymoma survival that were not mediated by the stage of disease at diagnosis, suggesting that male sex itself may be a risk factor for death after ependymoma diagnosis [10]. When looking at ependymoma outcomes generally, males have worse OS when compared to females with 5‐year OS of 71% as opposed to 78% in females [10]. Most epidemiologic studies lack methylation‐based subgroups, but there is evidence of sex differences in the distribution of these subgroups from published clinical case series [6].

DNA methylation helps regulate gene expression, but aberrant methylation can be associated with gene transcription modifications that are pathogenic [11]. Methylation differences based on sex are seen throughout the body in different organ systems [12]. Given the male excess in ependymoma incidence and death, along with our knowledge that methylation plays a role in the development of cancers, particularly in brain tumour formation and progression [12], we sought to identify sex differences in tumour methylation profiles within prognostic subgroups of ependymoma. Using publicly available data from Pajtler et al. (2015) [6], we identified sex differences in survival and methylation profiles and then validated the methylation findings in the Molecular Characterisation Initiative [13] using tumour data from individuals with ependymoma for some subgroups.

## 2 Materials and Methods

### 2.1 Data Source

Publicly available DNA methylation data from Pajtler et al. (2015) [6] were used for this analysis (GEO Series accession number GEO: GSE65362, date downloaded: 30 May 2021). There were 492 ependymoma samples from the initial diagnosis. Subgroups were included in the analysis if there were greater than 10 samples within the subgroup. Subgroups included in the analysis were PF‐EPN‐A (*n* = 238), PF‐EPN‐B (*n* = 52), PF‐SE (*n* = 34), SP‐EPN (*n* = 21), SP‐MPE (*n* = 26), ST‐SPN‐RELA (*n* = 87), ST‐TPN‐YAP1 (*n* = 13) and ST‐SE (*n* = 21). Already normalised (preprocessIllumina) methylated and unmethylated signal data were available. Clinical data for the Pajtler samples were obtained under Institutional Review Board approval at the University of Minnesota. Additional DNA methylation data (IDAT files, EPIC v1 array) for ependymoma tumours were obtained from Childhood Cancer Data Initiative (CCDI) Molecular Characterisation Inititative (MCI) [13]. The table of diagnosis and patient's included for both Pajtler and MCI can be found in Table 1.

**TABLE 1:** Clinical data and predicted molecular subtypes of ependymoma by predicted sex in Pajtler and COG MCI.

Pajtler	*N* = 492	Male, *N* = 302	Female, *N* = 190
Predicted subtypes, *n* (%)			
PF‐EPN‐A	238	154 (65)	84 (35)
PF‐EPN‐B	52	21 (40)	31 (60)
PF‐SE	34	26 (76)	8 (24)
SP‐EPN	21	13 (62)	8 (38)
SP‐MPE	26	14 (54)	12 (46)
ST‐EPN‐RELA	87	56 (64)	31 (36)
ST‐EPN‐YAP1	13	3 (23)	10 (77)
ST‐SE	21	15 (71)	6 (29)
Vital status, *n* (%)			
Alive	273	154 (56)	119 (44)
Death	83	56 (67)	27 (33)
Missing	136	92	44
Progression, *n* (%)			
Not progression	168	87 (52)	81 (48)
Progression	198	127 (64)	71 (36)
Missing	126	88	38
COG MCI	*N* = 103	Male, *N* = 58	Female, *N* = 45
Predicted subtypes, *n* (%)			
PF‐EPN‐A	45	23 (51)	22 (49)
PF‐EPN‐B	34	24 (71)	10 (29)
SP‐MPE	14	5 (36)	9 (64)
ST‐EPN‐RELA	10	6 (60)	4 (40)

*Note:* One case diagnosed with astrocytoma was excluded. Distributions of molecular subtypes of ependymoma in Pajtler and COG APEC14B1‐MCI were statistically significantly different by sex according to Fisher's exact test for count data with simulated *p* value (based on 2000 replicates).

### 2.2 Survival Analysis

Five‐year OS and PFS were estimated for males and females in all subgroups combined and within molecular subgroups of ependymoma. We defined OS as the time from the date of diagnosis of ependymoma to the date of the last follow‐up or date of death in months (*n* = 359), and PFS as the time from the date of diagnosis of ependymoma to the date of cancer progression in months (*n* = 365). We estimated 5‐year OS and PFS (95% confidence intervals) by sex in all subgroups combined and within molecular subgroups of ependymoma. Kaplan–Meier (KM) curves were constructed to identify sex differences in the 5‐year OS and PFS by sex in all subgroups combined and within molecular subgroups of ependymoma. The log‐rank *p* values were estimated to identify sex differences in 5‐year OS and PFS. We used R version 4.2.2 (2022‐10‐31 ucrt) and survival package survminor to implement survival analysis.

### 2.3 Quality Control and Sex Prediction

For each dataset, badly performing arrays (samples) were identified and removed using minfi's get QC function. Prenormalised methylated and unmethylated signals were used for the Pajtler dataset, while the MCI dataset was normalised using preprocess Funnorm. Probes containing known SNPs and probes with detection *p* values > 0.01 were removed from the dataset. Sex prediction was performed using minfi getSex on each dataset. As the Pajtler and MCI datasets came from different arrays, overlapping probes were selected for each dataset. Beta values for arrays (samples) and probes that passed QC and are present on both arrays were included in the final dataset.

### 2.4 Subgroup Prediction

Beta values for each dataset were combined, and study‐related batch effects were removed using limma removeBatchEffects(). The Pjalter dataset included subgroup information for each tumour and was used to create a random forest model to predict subgroups for the MCI dataset. Batch‐corrected beta values for the Pjalter dataset were divided into a training set (70% of the dataset) and a test set (30% of the dataset). Principal components (PCs) were calculated from the top 10,000 most variable probes. The top 10 PCs were used to train a random forest model (randomForest()) to predict subgroups from the training set. This model was then used to predict subgroups for the training set resulting in an OOB (out of bag) error of 0.75% (1 wrong call). The beta values for the probes included in the model were then isolated from the MCI dataset, and the top 10 PCs were calculated. These PCs were used as input for the model to predict subgroups for the MCI dataset.

### 2.5 Differential Methylation Analysis

All samples and probes that passed QC in the Pajtler dataset were used for differential methylation analysis using dmpFinder. DMP analysis was done between males and females within each subgroup. Significant DMPs had False discovery rate (FDR) < 0.05. DMPs were mapped to the nearest gene using the annotation information that comes with the array. Heatmaps were generated using the normalised beta values for significant DMPs.

### 2.6 Reactome Pathway Analysis

We identified biological pathways that had significant sex‐DMP in each subgroup of ependymoma. This was done using reactome pathway analysis. Pathways of interest were chosen if their FDR was 10% or less.

### 2.7 Immune Cell Profiling Based on Methylation Values

To assess immune cell composition within each tumour subgroup by sex and sex overall, we used the MethylCIBERTSORT package (version 0.2.0) [14] and CIBERSORTx (https://cibersortx.stanford.edu/) [15]. Beta values for reference probes were uploaded as percentages to CIBERSORTx. Using 1000 permutations without quartile normalisation, the data were analysed, and boxplots were created in R.

### 2.8 Validation Analysis

We validated the DMP results from Pajtler data in a linked dataset from the CCDI: MCI [16]. MCI samples were run on the Illumina Human Methylation EPIC array. Methylation data for cases were pulled on (date: 12 June 2024). The R package *minfi* was used to perform quality control (minfi plotQC) and sex prediction (minfi getSex) following the methods described in the section Quality Control and Sex Prediction. According to the bad sample cutoff equal to 10.5, samples with problematic probes that had lower median intensities and did not cluster were removed. Subgroups were removed if there were not at least 10 samples within the subgroup. Subgroups that remained in the analysis included: PF‐EPN‐A, PF‐EPN‐B, SP‐MPE and ST‐EPN‐RELA. A total of 103 samples were retained in the analysis (male: 58 (56%) vs. female: 45 (44%)). Two‐sample *t*‐tests were used to examine statistically significant differences in DNA methylation within molecular subgroups of ependymoma by sex, as we had done in the Pajtler data. FDR correction was performed for adjusting multiple comparisons, FDR cutoff used was < 0.05. Furthermore, an agreement between the results regarding sex differences in DNA methylation within molecular subgroups of ependymoma in the training and validation datasets was defined as the same direction of mean differences between the two sexes.

## 3 Results

There were 492 cases included in the discovery analysis: PF‐EPN‐A (*n* = 238) PF‐EPN‐B (*n* = 52), PF‐SE (*n* = 34), SP‐MPE (*n* = 26), SP‐EPN (*n* = 21), ST‐EPN‐RELA (*n* = 87), ST‐EPN‐YAP1 (*n* = 13) and ST‐SE (*n* = 21).

**FIGURE 1:**
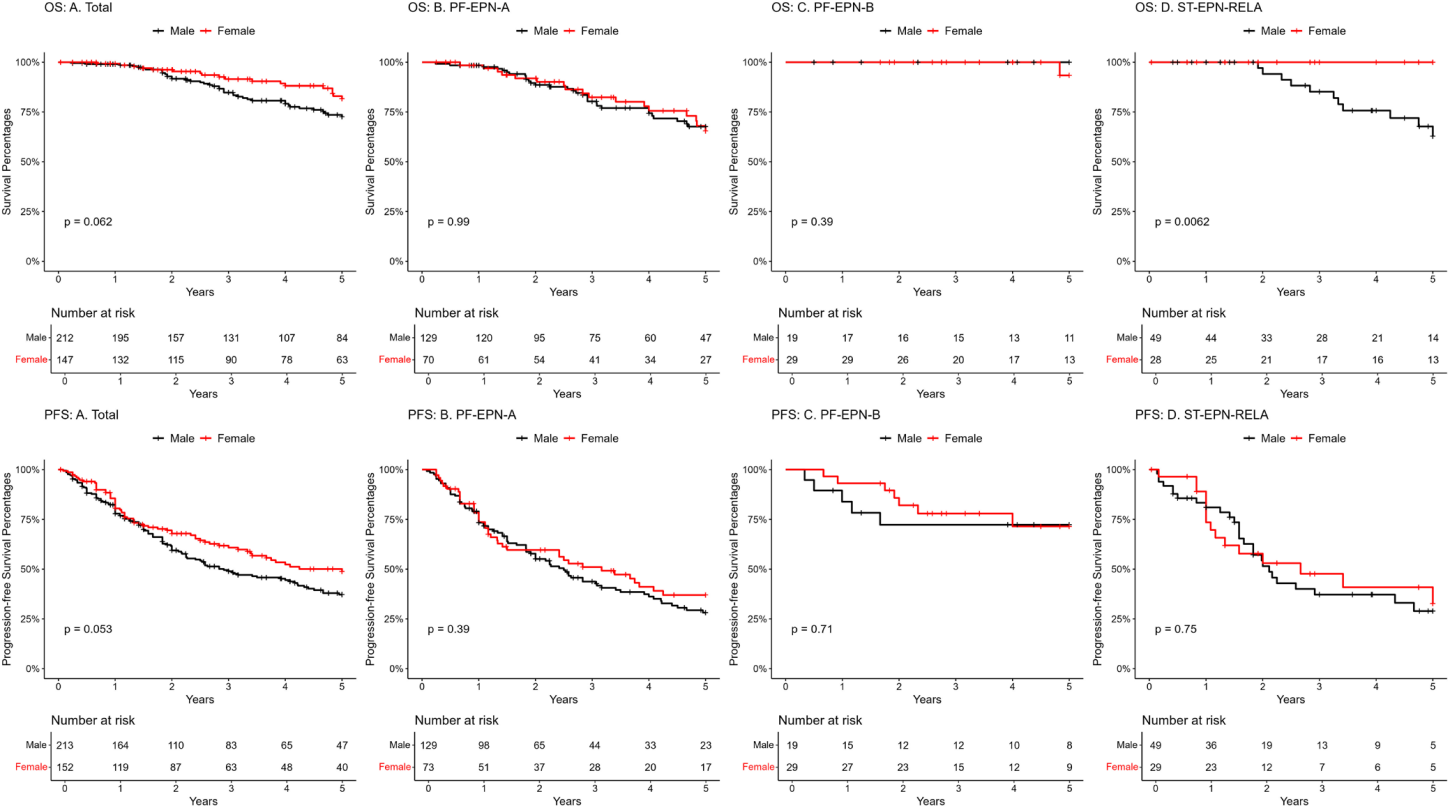
Kaplan–Meier survival curves showing five‐year overall survival (OS) and five‐year progression‐free survival (PFS) for those subgroups with less than 100% survival at 5 years by sex from the Pajtler data. (OS: A) Total, (OS: B) PF‐EPN‐A, (OS: C) PF‐EPN‐B, (OS: D) ST‐EPN‐RELA, (PFS: A) Total, (PFS: B) PF‐EPN‐A, (PFS: C) PF‐EPN‐B and (PFS: D) ST‐EPN‐RELA.

Females were detected as having better, but statistically insignificant 5‐year (82% vs. 73%) OS compared to males. Females also showed better and marginally significant 5‐year PFS overall than males (49% vs. 37%). One subgroup, ST‐EPN‐RELA, also showed significantly better 5‐year OS of females when compared to males (100% vs. 63%). Both females and males were reported 100% 5‐year OS in PF‐SE, SP‐EPN, SP‐MPE, SP‐SE and ST‐EPN‐YAP1. These results are found in Table 2 and Figure 1.

**TABLE 2:** 5‐Year overall survival and progression‐free survival estimates by sex in molecular subtypes of ependymoma.

Characteristic	5‐Year overall survival			5‐Year progression‐free survival			
	*N*	Event *N*	Probability (95% CI)	*N*	Event *N*	Probability (95% CI)	
Total							
Overall	359	60	76% (71%, 82%)	359	78	69% (63%, 76%)	
Predicted sex	359	60		359	78		
Male	212	42	73% (66%, 80%)	212	54	64% (56%, 73%)	
Female	147	18	82% (74%, 90%)	147	24	76% (68%, 86%)	
PF‐EPN‐A							
Overall	199	48	67% (59%, 75%)	199	57	60% (51%, 69%)	
Predicted sex	199	48		199	57		
Male	129	31	68% (59%, 78%)	129	39	57% (47%, 69%)	
Female	70	17	65% (53%, 81%)	70	18	64% (51%, 80%)	
PF‐EPN‐B							
Overall	48	1	96% (89%, 100%)	48	3	93% (85%, 100%)	
Predicted sex	48	1		48	3		
Male	19	0	100% (100%, 100%)	19	1	94% (83%, 100%)	
Female	29	1	93% (82%, 100%)	29	2	92% (83%, 100%)	
PF‐SE							
Overall	9	0	100% (100%, 100%)	9	0	100% (100%, 100%)	
Predicted sex	9	0		9	0		
Male	4	0	100% (100%, 100%)	4	0	100% (100%, 100%)	
Female	5	0	100% (100%, 100%)	5	0	100% (100%, 100%)	
SP‐EPN							
Overall	9	0	100% (100%, 100%)	9	0	100% (100%, 100%)	
Predicted sex	9	0		9	0		
Male	4	0	100% (100%, 100%)	4	0	100% (100%, 100%)	
Female	5	0	100% (100%, 100%)	5	0	100% (100%, 100%)	
SP‐MPE							
Overall	1	0	100% (100%, 100%)	1	0	100% (100%, 100%)	
Predicted sex	1	0		1	0		
Male	1	0	100% (100%, 100%)	1	0	100% (100%, 100%)	
Female	0	NA	NA	0	NA	NA	
ST‐EPN‐RELA							
Overall	77	11	76% (65%, 90%)	77	18	54% (39%, 76%)	
Predicted sex	77	11		77	18		
Male	49	11	63% (47%, 84%)	49	14	51% (34%, 76%)	
Female	28	0	100% (100%, 100%)	28	4	64% (38%, 100%)	
ST‐EPN‐YAP1							
Overall	10	0	100% (100%, 100%)	10	0	100% (100%, 100%)	
Predicted sex	10	0		10	0		
Male	1	0	100% (100%, 100%)	1	0	NA	
Female	9	0	100% (100%, 100%)	9	0	100% (100%, 100%)	
ST‐SE							
Overall	6	0	100% (100%, 100%)	6	0	100% (100%, 100%)	
Predicted sex	6	0		6	0		
Male	5	0	100% (100%, 100%)	5	0	100% (100%, 100%)	
Female	1	0	NA	1	0	NA	

Abbreviation: CI, confidence interval.

**FIGURE 2:**
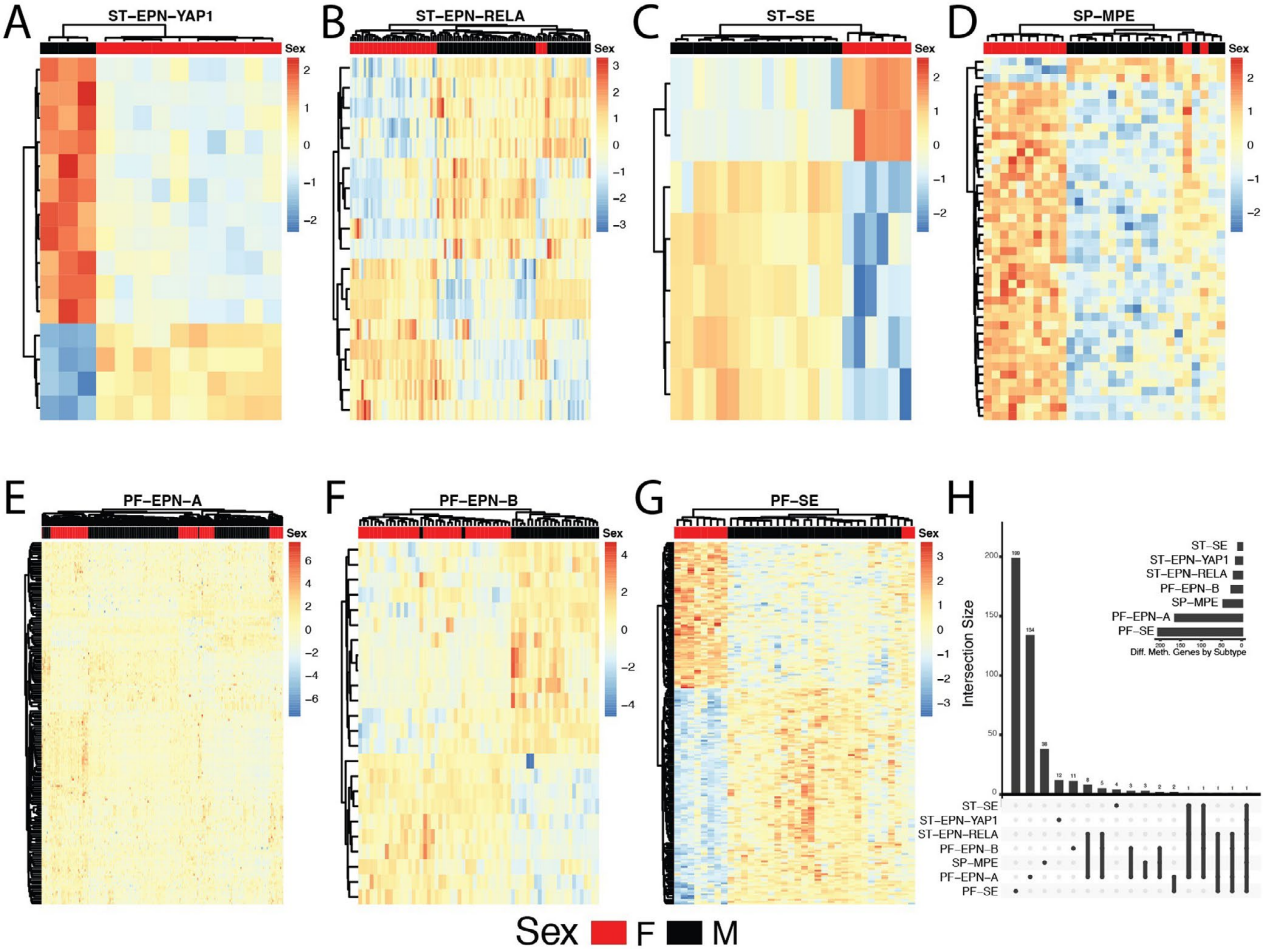
Heatmaps showing methylation levels (row‐scaled *b*‐values) of statistically significant differentially methylated positions (DMPs) by sex within ependymoma subgroups: (A) ST‐EPN‐YAP1, (B) ST‐EPN‐RELA, (C)ST‐SE, (D) SP‐MPE, (E) PF‐EPN‐A, (F) PF‐EPN‐B, (G) PF‐SE and (H) Upset plot showing unique and shared differentially methylated genes across ependymoma subgroups from the Pajtler data.

We observed statistically significant sex differences in DNA methylation within each included subgroup, except for SP‐EPN. PF‐SE had the highest number of DMPs by sex (*n* = 199), followed by PF‐EPN‐A (*n* = 164), SP‐MPE (*n* = 44), ST‐EPN‐RELA (*n* = 18), PF‐EPN‐B (*n* = 24), ST‐EPN‐YAP1 (*n* = 15) and ST‐SE (*n* = 7). Statistically significant DMPs by sex mapped to the gene RFTN1 in all the subgroups. Unsupervised hierarchical clustering of significant DMPs by sex within each subgroup was done. We found clustering was relatively strong by sex, but not by other characteristics as shown in the heat maps (Figure 2). PF‐EPN‐B, SP‐MPE, ST‐EPN‐YAP1 and ST‐SE each showed complete clustering by sex‐DMPs.

Pathways resulting from sex‐DMPs mapped to nearest gene for each subgroup are presented in Table 3. There were specific pathways identified from genes that were differentially methylated by sex in the following subgroups: PF‐EPN‐B, PF‐ SE, ST‐EPN‐RELA, ST‐EPN‐YAP1. Top pathways in PF‐EPN‐B include RNA polymerase transcription and promoter clearance as well as SMAD domain mutants in cancer. The top pathways in PF‐SE include FGFR2 signalling and activation. The top pathways in ST‐EPN‐RELA include mitochondrial and keratan sulfate biosynthesis. Many significant pathways from genes with sex DMPs were found in ST‐EPN‐YAP1 and include ERBB2 signalling, PI3K signalling, Interferon‐gamma and other cytokine signalling.

**TABLE 3:** Reactome pathway analysis (FDR < 10%) using genes that had sex‐DMPs identified within each subgroup from the Pajtler data. No pathways were identified for PF‐EPN‐A, ST‐SE and SP‐MPE.

Reactome pathway name	FDR
*PF‐EPN‐B*	
Mitochondrial biogenesis	0.09443772
RNA polymerase transcription	0.09443772
HIV transcription initiation	0.09443772
RNA polymerase II transcription initiation	0.09443772
RNA polymerase II HIV promoter escape	0.09443772
RNA polymerase II promoter escape	0.09443772
RNA polymerase II transcription initiation and promoter clearance	0.09443772
Loss of function SMAD4 in cancer	0.09443772
SMAD4 MH2 domain mutants in cancer	0.09443772
SMAD2/3 MH2 domain mutants in cancer	0.09443772
*PF‐SE*	
Signalling by FGFR2 IIIa TM	0.02478109
FGFR2 mutant receptor activation	0.02478109
Signalling by FGFR2 in disease	0.08176046
*ST‐EPN‐RELA*	
Mitochondrial biogenesis	0.09483678
Keratan sulfate biosynthesis	0.09483678
*ST‐EPN‐YAP1*	
GRB7 events in ERBB2 signalling	0.00282658
Downregulation of ERBB2:ERBB3 signalling	0.00463631
Constitutive signalling by aberrant PI3K in cancer	0.00463631
ERBB2 activates PTK6 signalling	0.00463631
ERBB2 regulates cell motility	0.00463631
PI3K events in ERBB2 signalling	0.00516862
PI3K/AKT signalling in cancer	0.00573294
PI5, PP2A, and IER3 Regulate PI3K/AKT signalling	0.00573294
Negative regulation of the PI3K.AKT network	0.00573294
Signalling by ERBB2 TMD/JMD mutants	0.00573294
Signalling by ERBB2 KD mutants	0.00639273
SHC1 events in ERBB2 signalling	0.00639273
Downregulation of ERBB2 signalling	0.00639273
Signalling ERBB2 in cancer	0.00639273
Signalling by ERBB2	0.01909767
Signalling PTK6	0.02077429
Signalling by nonreceptor tyrosine kinases	0.02077429
Signalling by ERBB4	0.02290672
PIP3 activates AKT signalling	0.03364767
Intracellular signalling by second messengers	0.04900415
Formyl peptide receptors bind formyl peptides and many other ligands	0.05411417
Calcitonin‐like ligand receptors	0.05411417
PI3K events in ERBB4 signalling	0.07362005
OAS antiviral response	0.07362005
Interferon alpha/beta signalling	0.07703115
Diseases of signal transduction by growth factor receptors and second messengers	0.07703115
SHC1 events in ERBB4 signalling	0.07703115
GRB2 events in ERBB2 signalling	0.07703115
TFAP2 (AP‐2) family regulates the transcription of growth factors and their receptors	0.07703115
Interleukin‐4 and Interleukin‐13 signalling	0.07867710
Regulation of TP53 activity through methylation	0.07867710
Intrinsic pathway of fibrin clot formation	0.07867710
Interferon‐gamma signalling	0.07867710
PIWI‐interacting RNA (piRNA) biogenesis	0.07867710
Long‐term potentiation	0.07867710
ESR‐mediated signalling	0.07867710
Cytokine signalling in immune system	0.08308865
DNA damage recognition in GG‐NER	0.09674191

**FIGURE 3:**
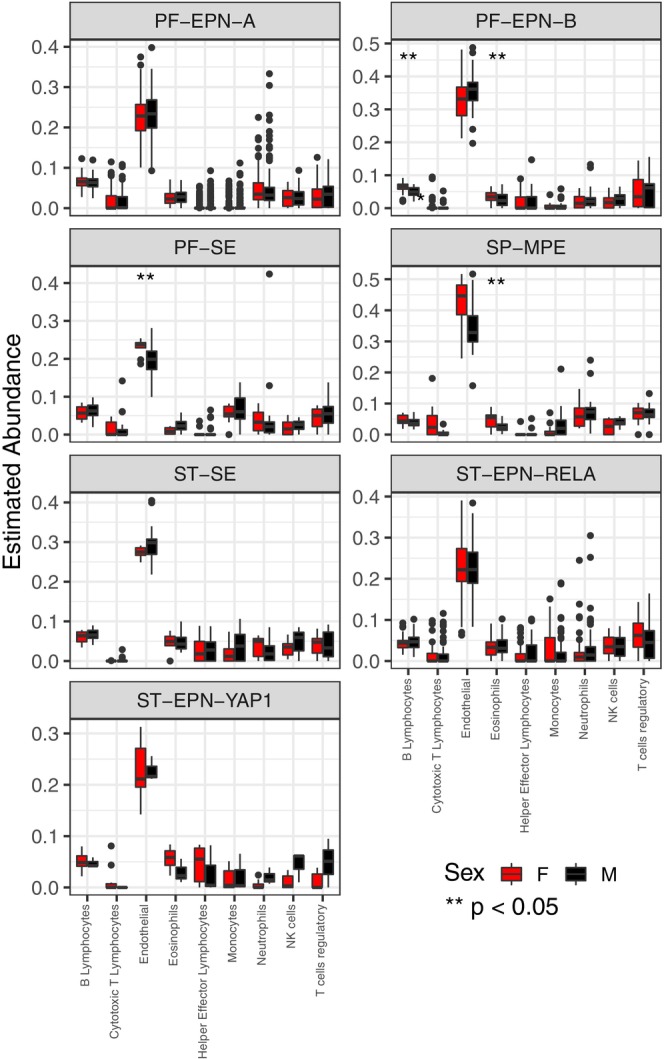
MethylCIBERSORT stromal signature matrix within ependymoma subgroups from the Pajtler data. Boxplots compare ependymoma subgroup means for each cell type. Significance was calculated using pairwise Wilcoxon Rank Sum tests. *p* values (Boxplots here compare female (red) and male (black) means for each cell type in each ependymoma subgroup as labelled. ** *p* value used for significance was ≤ 0.05.

As there is a complex link between the role of immune cells in tumour development and progression [17], using the methylation data, we estimated immune cell composition between subgroups and between sexes within subgroups. There were statistically significant differences in immune cell composition within the tumour based on a molecular subgroup (Figure S1). There were also significant differences in immune cell type present between sexes within subgroups as well. In PF‐EPN‐B, there were differences in B lymphocyte and eosinophil composition. In PF‐SE, there was a difference in cytotoxic T lymphocytes. In SP‐MPE, there was a difference in eosinophil composition (Figure 3).

**FIGURE 4:**
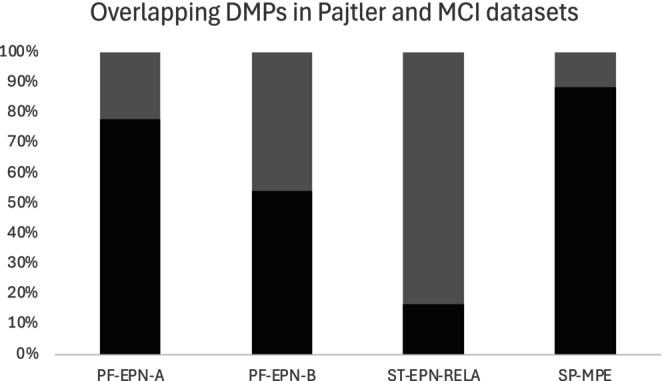
Percent of overlapping DMPs found within Pajtler and MCI datasets. Here, 100% shows the sex DMPs in each subgroup in the Pajtler dataset, and the grey bar indicates the overlapping sex DMPs that were also statistically significantly differentially (FDR < 0.05) methylated in the MCI dataset in each subgroup.

In the validation MCI dataset (*n* = 103), sex differences in DNA methylation were assessed in four molecular subgroups of ependymoma that were the exact same molecular subgroups with statistically significant sex differences in DNA methylation in the Pajtler dataset. The FDR‐adjusted *p* values indicated that these four molecular subgroups of ependymoma in MCI also had statistically significant differences in DMPs which overlapped with those seen in the Pajtler dataset between males and females for the following subgroups: PF‐EPN‐A (*n* = 36), PF‐EPN‐B (*n* = 11), SP‐MPE (*n* = 5) and ST‐EPN‐RELA (*n* = 15). This can be seen in Figure 4.

## 4 Discussion

From 492 primary ependymoma samples, we identified sex differences in 5‐year OS within all cases and in ST‐EPN‐RELA with females having better OS than males. We also identified sex differences in methylation within each subgroup, excluding SP‐EPN. PF‐SE had the higher number of DMPs by sex (*n* = 283), followed by PF‐EPN‐A (*n* = 240), SP‐MPE (*n* = 56), ST‐EPN‐RELA (*n* = 36), PF‐EPN‐B (*n* = 25), ST‐EPN‐YAP1 (*n* = 18) and ST‐SE (*n* = 10). In the MCI validation cohort, statistically significant sex differences in DNA methylation were identified in four ependymoma subgroups: PF‐EPN‐A (*n* = 36), PF‐EPN‐B (*n* = 11), SP‐MPE (*n* = 5) and ST‐EPN‐RELA (*n* = 15). These four subgroups were the only subgroups that had greater than 10 samples and thus were the only subgroups included in the analysis in the validation cohort. Unsupervised hierarchical clustering was not driven by other clinical factors, suggesting that there is a true sex difference in DMPs independent of clinical factors.

There is an increased incidence of ependymoma in males overall [9]. When looking at incidence and survival outcomes based on sex, there is often no distinction between subgroups, particularly in epidemiologic analyses as these data are lacking. In our sample set, we observed a male excess in diagnoses in all subgroups except PF‐EPN‐B and ST‐EPN‐YAP1. There are noted worse outcomes in males compared to females in ependymoma historically [10, 18]. We did see worse overall survival in males than in females, although this was marginally nonsignificant. We also saw worse PFS in males than in females overall. Within subgroups, there was also worse OS seen in males compared to females in one subgroup, ST‐EPN‐RELA, which comprises 70% of paediatric supratentorial ependymoma cases [4].

Concerning our investigation into sex differences in methylation, there was one gene, RFTN1, shared between all subgroups that had sex DMPs. The RFTN1 gene has been associated with T‐cell binding and T‐cell activation [19] and other T‐cell signalling pathways through the T‐cell receptor [20]. With increased proportions of CD8^+^ and CD4^+^ T cells in samples with higher RFTN1 expression, and subsequently more chemotactic factors, RFTN1 may enhance antitumour immunity [19]. Although not much is known about the expression of RFTN1 in brain tumours, there is information in other cancer types. RFTN1 is known to increase likelihood of progression in gastric cancer with higher expression leading to increased proliferation and decreased apoptosis [21]. RFTN1 expression is increased in skin cancer development as well [22]. It is downregulated in breast cancer [23] and ovarian cancer [24]. Increased RFTN1 expression is noted to be a negative prognostic factor in renal cancers [25]. Overactive T cells from increased RFTN1 expression within the brain could lead to neuroinflammation and may cause altered immune regulation as well as increased proliferation and decreased apoptosis of tumour cells. With continued understanding and further research with this gene, it could become a potential diagnostic biomarker or therapeutic target for certain cancer types. RFTN1 is also associated with double‐stranded RNA binding, B‐cell receptor signalling and toll‐like receptor signalling [25, 26].

We were able to classify reactome pathways from the sex‐DMPs that were mapped to genes in each subgroup of ependymoma (Table 3). Significant pathways in the PF‐EPN‐B subgroup were RNA polymerase transcription and promoter clearance as well as SMAD domain mutants in cancer. SMAD mutations are associated with pancreatic and other GI cancers [27]. Mutations in the MH1 domain of SMAD can affect DNA binding [27]. Alterations to DNA binding proteins affect transcription factors and are associated with the development and progression of certain cancers. These transcription factors can then be targets for therapeutic intervention [28]. Whereas mutations in the MH2 region can interfere with TGF‐beta signalling [29]. Alteration to TGF‐beta signalling can lead to tumour development and progression as well [30]. Overexpression of TGF‐beta in brain tumours like ependymoma could lead to increased cell proliferation as well as increased tumour invasion and angiogenesis. Top pathways in PF‐SE include FGFR2 signalling and activation. FGFR is associated with high‐risk paediatric ependymomas [31]. FGFR‐targeted therapies are another promising option for future studies in paediatric ependymoma [32] and may show sex‐specific effects through an interaction with the underlying DNA methylation status of the gene.

Top pathways identified from sex‐DMP genes in ST‐EPN‐YAP1 include ErbB2 signalling as well as PI3K signalling. ErbB2 pathway is a tyrosine kinase pathway that is involved with cell growth and differentiation [27]. Receptors for this pathway are found in many ependymoma samples in children, with higher expression causing increased proliferation and subsequently worse outcomes [33]. Lapatinib is ErbB1 and ErbB2 inhibitors that can cross the blood–brain barrier; however, it was not specifically tested in ependymoma in vitro [33]. There was a phase 1 trial looking at lapatinib in refractory paediatric brain tumours which showed it was well‐tolerated [34], but subsequent studies found no significant outcome differences between this drug and standard therapy [35, 36]. The PI3K pathway is associated with growth factors and induces cell division and apoptosis. In ependymoma, there is more expression of genes in this pathway in supratentorial tumours as opposed to posterior fossa or spinal ependymomas and higher expression of proteins in this pathway can be used as a marker of worse PFS [37]. There has not been information about PI3K expression by ependymoma subgroups over tumour locations. This could also be a potential therapeutic target in this patient population. The pathways associated with the RFTN1 gene do not appear to be associated with top pathways in each subgroup, and thus, the functional significance of the pathways within each subgroup remains unclear.

There are known sex differences in brain tumours and genomic differences discussed in the literature [38, 39]. Through methylation differences and hormonal changes, there is a difference in the tumour environment and tumorigenesis among different sexes [38]. In our study, we saw the highest number of sex‐DMPs in PF‐SE which are often seen in adults with overall good outcomes [40]. The next highest number of sex‐DMPs was seen in PF‐EPN‐A which is most commonly seen in children with a male predominance and overall poor survival [6]. It remains unclear whether epigenetic changes associated with sex differences are driving outcomes found in the epidemiologic literature.

While we have a large discovery dataset and a validation dataset for ependymoma, which is a rare paediatric brain tumour, there are limitations to this study. We were not able to obtain survival data for the validation dataset from MCI [13]. We also were unable to obtain enough cases in the additional cohort to validate all the different subgroups of ependymoma. This study is not a population‐based study, but rather a clinical cohort, and thus, it may not be representative of the distribution of ependymoma subgroups in the general population as the genomic profiling of these tumours requires tumour tissue availability, which may be differently distributed in a population‐based setting where surgery is not an option for all individuals with ependymoma depending on tumour location and size. There may be other differences between sex including other clinical criteria, risk factors, or response to therapy which may contribute to potential differences seen in outcomes between males and females, but this information was not available for evaluation within our study.

In conclusion, we were able to identify differences in DMPs within subgroups of ependymoma with PF‐SE having the highest number of DMPs, closely followed by PF‐EPN‐A. We did find overall better outcomes in females overall and within ST‐EPN‐RELA. There was one sex differentially methylated gene (RFTN1) common to all subgroups, and there were pathway differences among four of the subgroups. Many of the identified pathways are known to have associations with ependymoma and are potential options for future therapeutic targets in the most high‐risk cases.

## Author Contributions

**Shelby Mestnik:** formal analysis (supporting), writing – original draft (lead). **Natali Sorajja:** conceptualization (supporting), data curation (lead), investigation (lead), writing – original draft (supporting), writing – review and editing (equal). **Zhanni Lu:** formal analysis (equal), writing – review and editing (equal). **Lauren J. Mills:** formal analysis (equal), writing – review and editing (equal). **Lindsay Williams:** conceptualization (lead), data curation (supporting), investigation (supporting), writing – review and editing (equal).

## Conflicts of Interest

The authors declare no conflicts of interest.

## Supporting information


Figure S1.

## Data Availability

Data is publicly available through Pajtler et al. GEO Series accession number GSE65362 as well as through the molecular characterization initiative (MCI).
