# Supplementary figures and images for: Sex Differences in Ependymoma Methylation by Methylation‐Defined Subgroup
Source: J Cell Mol Med. 2024 Dec 16;28(24):e70286. doi: 10.1111/jcmm.70286 (PMC11647990; doi:10.1111/jcmm.70286)

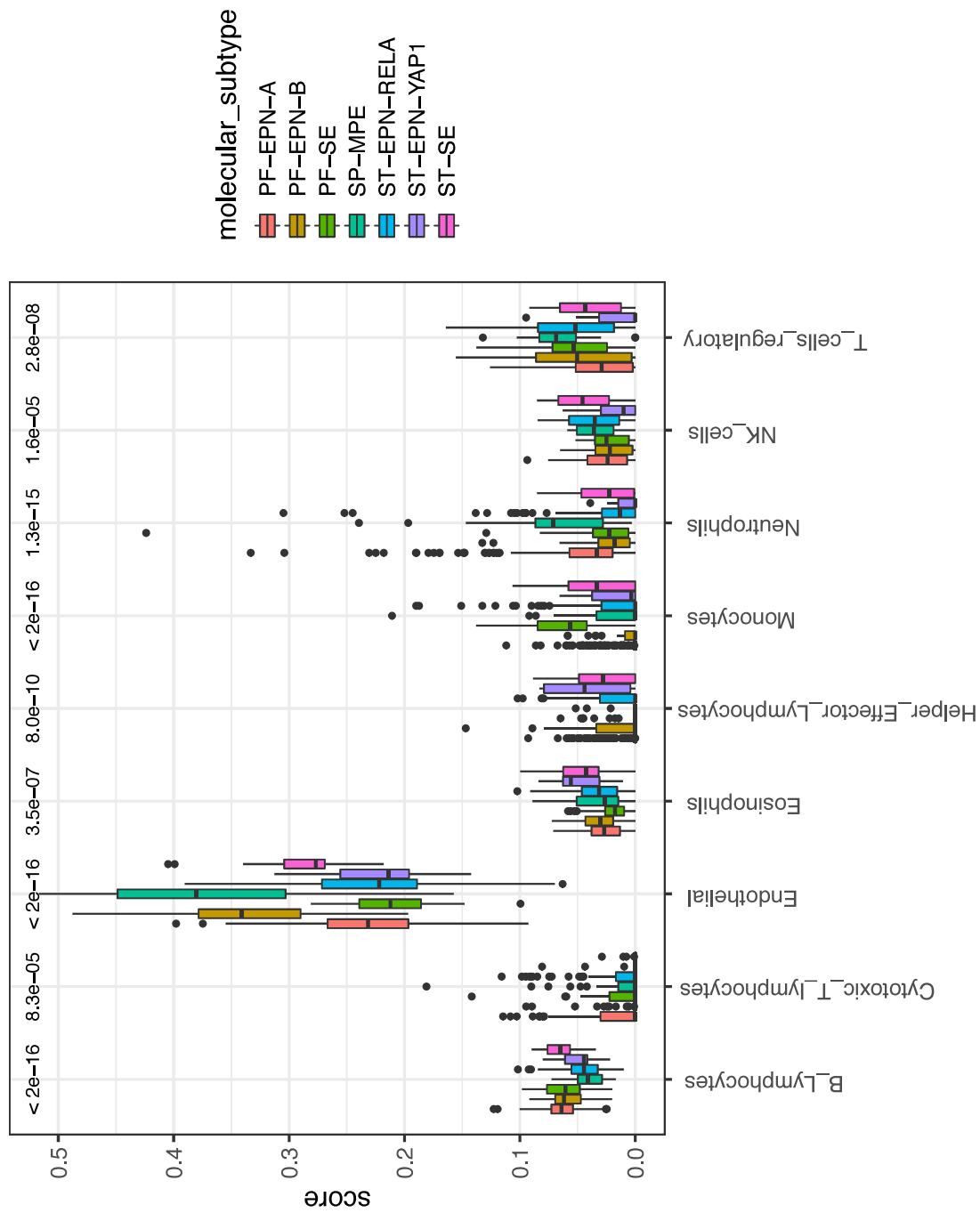

Supplement: Supplementary file 1 — Figure S1. [file JCMM-28-e70286-s001.pdf]
